# Do Different Two-Dimensional Camera Speeds Detect Different Lower-Limb Kinematics Measures? A Laboratory-Based Cross-Sectional Study

**DOI:** 10.3390/jcm14051687

**Published:** 2025-03-02

**Authors:** Abdulaziz Rsheed Alenzi, Msaad Alzhrani, Ahmad Alanazi, Hosam Alzahrani

**Affiliations:** 1 Department of Physical Therapy, Move Comprehensive Sports Medicine Center, Riyadh 11564, Saudi Arabia; a.alenzi@move.med.sa; 2 Department of Physical Therapy and Health Rehabilitation, College of Applied Medical Sciences, Majmaah University, Majmaah 15341, Saudi Arabia; m.alzhrani@mu.edu.sa (M.A.); aalanazi@mu.edu.sa (A.A.); 3 Department of Physical Therapy, College of Applied Medical Sciences, Taif University, Taif 21944, Saudi Arabia

**Keywords:** biomechanics, camera, lower extremity, sports injuries, two-dimensional

## Abstract

**Background/Objectives:** Football poses a high risk of sustaining lower-limb injuries, particularly anterior cruciate ligament (ACL) injuries, owing to the frequent jumping and landing movements. Identifying risk factors for these injuries is crucial to successful prevention. Two-dimensional (2D) video analysis is a commonly employed tool for assessing movement patterns and determining injury risk in clinical settings. This study aims to investigate whether variations in the camera frame rate impact the accuracy of key angle measurements (knee valgus, hip adduction (HADD), and lateral trunk flexion (LTF)) in male football players during high-risk functional tasks such as single-leg landing and 45° side-cutting. **Methods:** This laboratory-based cross-sectional study included 29 football players (mean (SD) age: 24.37 [3.14] years). The frontal plane projection angle (FPPA), HADD, and LTF during single-leg landing and side-cutting tasks were measured using two different camera frame rates: 30 frames per second (fps) and 120 fps. The 2D kinematic data were analyzed using Quintic Biomechanics software. **Results:** Significant differences in FPPA scores during single-leg landing were observed between the 30 fps and 120 fps for both the dominant (mean difference = 2.65 [95% confidence interval [CI]: 0.76–4.55], *p* = 0.008) and non-dominant leg (3.53 [1.53–5.54], *p* = 0.001). Additionally, the FPPA of the right leg during the side-cutting task showed significant differences (2.18 [0.43–3.93], *p* = 0.016). The LTF of the right leg during side-cutting displayed a significant variation between frame rates (−2.69 [−5.17–−0.22], *p* = 0.034). No significant differences in HADD were observed. **Conclusions:** Compared with a 30 fps camera, a high-speed (120 fps) camera demonstrated a superior performance in delivering accurate kinematic assessments of lower-limb injury risk factors. This improved precision supports injury screening, rehabilitation monitoring, and return-to-play decision-making through determining subtle biomechanical deficits crucial for lower-limb injury prevention and management.

## 1. Introduction

Football remains the most widely played sport globally [1], with more than 200 million registered members across 203 countries under the Federation of International Football Association (FIFA) [2]. Sports injuries account for up to 19% of all severe cases treated in emergency departments, with knee and ankle injuries being the most prevalent [3]. Despite its global popularity, the risk of injury is higher in football, owing to activities involving frequent jumping and landings [3].

Injuries are more frequent during competitive play than during training sessions. A global review of football-related injuries indicated that muscle strains, ligament sprains, and fractures are more prevalent among professional players, particularly lower-extremity injuries such as those in the ankle and knee [4]. Anterior cruciate ligament (ACL) injuries are the most serious globally, with an estimated 120,000 cases reported annually in the United States [5]. ACL injuries commonly occur in noncontact scenarios, such as rapid directional shifts or improper landings, often driven by biomechanical factors such as excessive knee valgus, tibial rotation, and greater joint loading [6]. Single-leg landing and side-cutting tasks are frequently utilized to evaluate ACL injury risk due to their ability to replicate these mechanisms of injury. Single-leg landings simulate unilateral loading scenarios, stressing neuromuscular control and stability under high joint stress, while side-cutting movements replicate dynamic, multiplanar forces experienced during quick directional changes, both of which are strongly associated with ACL strain [7,8,9].

Given the serious physical and career implications of these injuries in athletes, identifying and understanding the potential risk factors is crucial for prevention. Movement screening during high-risk activities is an effective method for assessing the risk of injury. Several studies have investigated approaches for identifying and predicting possible injury risks [10,11]. Although three-dimensional (3D) motion analysis remains the gold standard for movement evaluation, providing precise and reliable measurements of kinetic and kinematic variables during lower-limb motion [12], high cost, time consumption, and spatial requirements often limit its application in field settings or routine clinical practice [13].

To overcome these limitations, two-dimensional (2D) video analysis has been developed as a feasible and cost-effective alternative for evaluating dynamic movements [14]. When combined with basic video cameras and software, 2D analysis can provide valuable kinematic data, but with less precision than 3D systems. Nonetheless, 2D analysis is a reliable method for assessing lower-limb kinematics, particularly in the context of dynamic sports actions such as landing and jumping [14,15,16]. Key factors influencing the accuracy of 2D video analysis include the sampling frequency of the camera, measured in frames per second (fps), and pixel resolution, which substantially impacts the quality and precision of the recorded data [17].

The existing literature demonstrates wide variability in frame rate selection for human motion studies, with restricted justification for these choices [15,16,18,19,20]. For example, lower frame rates (≤60 fps) may inadequately sample high-velocity actions (e.g., rapid direction changes, or sprinting), leading to the temporal underestimation of peak joint angles [21]. Conversely, higher frame rates (≥120 fps) impose computational burdens without necessarily enhancing accuracy in slower movements, emphasizing a need for task-specific optimization [22]. In football, where movements such as 45° side-cuts and single-leg landings impose abrupt multidirectional loads on the knee and hip joints, suboptimal frame rates may mask critical kinematic events (e.g., lateral trunk flexion (LTF) or transient valgus collapse), thereby compromising injury risk assessments [23,24].

Despite these concerns, no prior research has systematically explored the association between frame rate and measurement error for football-specific tasks. This gap is particularly consequential given the growing reliance on 2D video for coaching, injury screening, and biomechanical feedback in professional football leagues, where 3D systems are financially inaccessible [22]. This study hypothesized that different frame rates (e.g., 30 fps vs. 120 fps) could result in significant differences in the captured angles of knee valgus, hip adduction, and LTF during single-leg landing and 45° side-cutting tasks. Therefore, this study aimed to determine the impact of camera frame rate variations on the measurement of the key angles (knee valgus, hip adduction, and LTF) in male football players during high-risk functional tasks (single-leg landing and 45° side-cutting).

## 2. Methodology

### 2.1. Study Design

This laboratory-based cross-sectional study was conducted in Riyadh, Saudi Arabia. This study adhered to the “Strengthening the Reporting of Observational Studies in Epidemiology (STROBE)” guidelines. The study protocol was approved by the Research Ethics Committee of Majmaah University (Application No. MUREC-Jan.28/COM-2021/21-3, approval date on 28 January 2021). Data collection was performed in March 2021.

### 2.2. Participants

This study included recreational male football players aged 18–30 years who played 3–5 times per week. Players with a history of lower extremity injury within the last 6 months that resulted in more than 7 days of lost time and those who had undergone ACL reconstruction were excluded. These exclusion criteria were implemented to reduce potential confounding variables that could impact kinematic measurements. Lower extremity injuries or previous ACL reconstruction may change movement patterns, neuromuscular control, and biomechanical responses, probably skewing the results. By excluding these participants, the study aimed to ensure including a homogenous sample, allowing for a more precise and accurate assessment of the influence of camera frame rate on kinematic measurements without the influence of other compromising factors.

Data collection was performed over 2 full days in Riyadh, Saudi Arabia. Participants were recruited using the databases of various local football teams during this period.

### 2.3. Setting

The study was conducted in a laboratory setting. Upon arrival at the recruitment area, the participants passed through a series of stations. At the first station, the participants completed a general information form, signed a research consent form, and received an overview of the project. Participants’ height and weight were measured at the second station. At the third station, a therapist applied reflective marks to the participants. The experiments were conducted at the last station. Four skilled therapists were assigned to manage the stations.

### 2.4. Instrumentation

#### 2.4.1. Nikon 250 Z Mount Camera

Two digital cameras with interchangeable lenses Nikon Z 250 (Nikon Corp., Tokyo, Japan) were used. Each camera had an effective pixel count of 20.9 million and a 23.5 × 15.7 mm complementary metal-oxide-semiconductor image sensor. The cameras recorded videos at multiple resolutions, including 5568 × 3712, 4176 × 2784, and 2784 × 1856 pixels; the frame rate of one camera was set to 30 fps and the other was set to 120 fps. To ensure consistency between the two frame rates, a calibration procedure was conducted before data collection. The cameras were placed at identical angles and distances relative to the capture area to provide a consistent field of view. Lens distortion correction was employed to account for any optical aberrations, and a static calibration frame was utilized to provide a shared referenced system for both cameras. Furthermore, the cameras were synchronized to maintain a proper temporal alignment of the recordings, reducing any potential differences in motion tracking between both the 30 fps and 120 fps conditions.

#### 2.4.2. Reflective Marker Placement

Before data collection, reflective markers were affixed to the participants’ anterior superior iliac spine (ASIS), midpoint of the femoral condyles (to estimate the knee joint center), and midpoint of the lateral malleolus (to estimate the ankle joint center). The joint medians were determined using a standard measuring tape.

### 2.5. Functional Tasks

#### 2.5.1. Single-Leg Landing Task

The participants were instructed to step down (straight downwards) from a 30 cm step box and lean forward. They were also instructed to maintain a single-leg stance on the opposite leg before moving forward and landing on a pre-designated marking connected to the landing leg, while ensuring that the non-landing leg did not contact the surface. A designated therapist confirmed that each participant successfully completed five trials while maintaining balance for 3 s in each trial. Assessments were conducted on both the dominant and non-dominant legs.

#### 2.5.2. Side-Cutting 45° Task

The participants completed five trials of a side-step cutting maneuver. They were instructed to run 5 m, place their right foot on a marked spot on the floor, and then move left. The cones were placed at 35° and 55° from the original progression direction to guide the participants to cut at a 45° angle. The practice trials helped to familiarize the participants with the method and instrumentation. The cameras were positioned in the frontal plane, 2 m in front of the landmark. The task was performed exclusively using the right leg.

### 2.6. Measuring the Angles

#### 2.6.1. Frontal Plane Projection Angle (FPPA)

The FPPA is defined as the angle between two lines: one connecting the markers from the proximal thigh to the knee joint and the other from the knee joint down to the ankle at maximal knee flexion. A positive FPPA score indicates knee valgus, i.e., the knee moves inward toward the body’s midline, with the knee marker positioned medial to the line connecting the ankle and thigh markers; a negative FPPA value indicates knee varus [18].

#### 2.6.2. Hip Adduction Angle (HADD)

To calculate the HADD angle for the right limb, a line was drawn from the left ASIS to the right ASIS, and another line from the right ASIS to the marker positioned at the midpoint of the right knee joint. For the left limb, the process was reversed with a line from the right ASIS to the left ASIS and another line from the left ASIS to the left knee midpoint marker. The standard 90° alignment between the ASIS and femoral lines was used as the 0° position, with a positive value suggesting HADD and a negative value indicating hip abduction [19].

#### 2.6.3. Lateral Trunk Flexion (LTF) Angle

The LTF was measured as the angle between the vertical line drawn at the ipsilateral ASIS and the line connecting the ipsilateral ASIS to the manubrium sterni. A smaller LTF indicated a greater LTF toward the supporting leg. The LTF was negative when the manubrium sterni was lateral to the ipsilateral ASIS [18].

### 2.7. Two-Dimensional Video Analysis

The 2D video analysis used two camera systems operating at different frame rates (30 and 120 fps) to record participants performing the same procedures. The cameras were mounted on a tripod positioned 2 m apart at a height of 0.8 m, perpendicular to the frontal plane of motion. Both cameras were aligned at the same vertical angle, and recording was performed simultaneously. Participants were instructed to complete five trials of single-leg landing and side-cutting tasks on both their dominant and non-dominant legs, and the data were recorded. A single rater analyzed the 2D kinematic data using Quintic Biomechanics software version 33 (Quintic, Sutton Coldfield, West Midlands, UK), extracting key metrics (FPPA, HADD, and LTF) relevant to this study.

### 2.8. Statistical Analysis

The normality of the sample data distribution was assessed using the Shapiro–Wilk test. A paired-sample *t*-test was performed to evaluate the differences between the 30 fps and 120 fps results for all outcome measures. The *p*-values were derived from the paired t-test, with α = 0.05, indicating statistical significance. Effect sizes were computed using Cohen’s d and were interpreted as small (d = 0.2), medium (d = 0.5), and large (d ≥ 0.8) [25]. Data were analyzed using SPSS software version 27.0 (IBM Corp., Armonk, NY, USA).

## 3. Results

This study included 29 male recreational football players (mean age: 24.37 [SD 3.14]). The demographic data of the participants are presented in Table 1.

**Figure 1:**
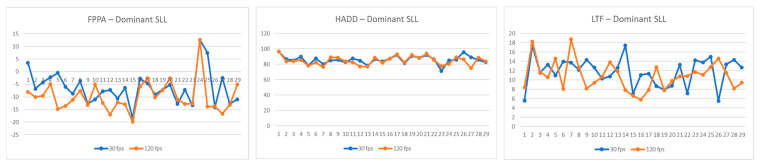
Comparison of frontal plane projection angle (FPPA), hip adduction angle (HADD), and lateral trunk flexion (LTF) during dominant-leg single-leg landing (SLL) at 30 fps and 120 fps, for each participant. Abbreviations: FPPA, frontal plane projection angle; HADD, hip adduction angle; LTF, lateral trunk flexion.

**Figure 2:**
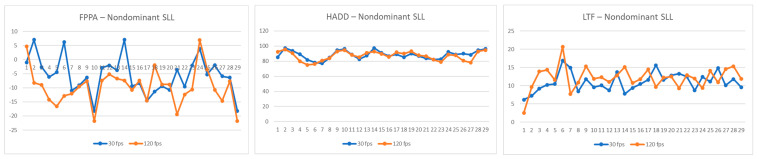
Comparison of frontal plane projection angle (FPPA), hip adduction angle (HADD), and lateral trunk flexion (LTF) during non-dominant leg single-leg landing (SLL) at 30 fps and 120 fps, for each participant. Abbreviations: FPPA, frontal plane projection angle; HADD, hip adduction angle; LTF, lateral trunk flexion.

**Figure 3:**
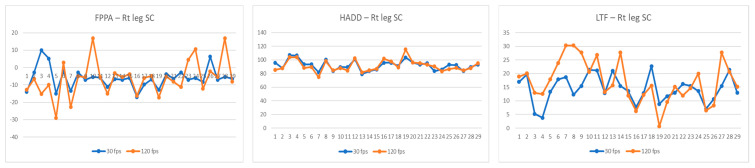
Comparison of frontal plane projection angle (FPPA), hip adduction angle (HADD), and lateral trunk flexion (LTF) during right-leg side-cutting (SC) at 30 fps and 120 fps, for each participant. Abbreviations: FPPA, frontal plane projection angle; HADD, hip adduction angle; LTF, lateral trunk flexion; SC, side-cutting.

Comparative values of the FPPA, HADD, and LTF recorded by the 30 fps and 120 fps cameras for the legs tested during the single-leg landing and side-cutting tasks are listed in Table 2 (and Figure 1, Figure 2 and Figure 3). Significant differences in FPPA scores were observed between the 30 fps and 120 fps camera conditions for both the dominant (mean difference = 2.65 [95% CI: 0.76–4.55], *p* = 0.008, d = 0.61) and non-dominant legs (mean difference = 3.53 [95% CI: 1.53–5.54], *p* = 0.001, d = 0.70) during the single-leg landing task, with higher FPPA values recorded by the 120 fps camera. Additionally, the FPPA of the right leg during the side-cutting task was significantly different between the 30 fps and 120 fps cameras, where the 120 fps camera recorded a greater FPPA (mean difference = 2.18 [95% CI: 0.43–3.93], *p* = 0.016, d = 0.40). Furthermore, the LTF during the side-cutting task revealed significant variations between the 30 fps and 120 fps cameras, with the 120 fps camera showing greater LTF values (mean difference = −2.69 [95% CI: −5.17–−0.22], *p* = 0.034, d = −2.20).

For the other angles, no significant differences were found between the 30 fps and 120 fps cameras.

## 4. Discussion

This study is the first to investigate the impact of different camera frame rates on FPPA, HADD, and LTF measurements in male football players during specific biomechanical tasks. The findings demonstrated significant differences in FPPA scores during both the single-leg landing and side-cutting tasks between the 30 fps and 120 fps camera rates, with the higher frame rate (120 fps) consistently recording higher FPPA values. Furthermore, a significant difference was observed in the LTF during the side-cutting task; the 120 fps camera consistently outperformed the 30 fps camera. However, no significant differences in HADD angles were observed between the two camera frame rates.

The findings revealed that higher frame rates, such as 120 fps, provide greater precision in biomechanical measurements, particularly for dynamic tasks such as single-leg landing and side-cutting. The ability of the 120 fps camera to capture higher FPPA values than that of the 30 fps camera suggests that lower frame rates may underestimate knee valgus during rapid deceleration or directional changes [24]. This potential underestimation has important clinical implications as knee valgus is a crucial factor in assessing the risk of ACL injuries. Precise assessment of these angular deviations is vital for developing effective injury prevention and rehabilitation strategies, particularly in sports such as football that involve frequent high-speed directional changes.

Comparing these results with those of previous research is challenging, owing to the lack of studies specifically examining the impact of different camera frame rates on lower-limb kinematics. Furthermore, there are no standardized guidelines for determining the optimal frame rate for 2D motion analysis of the lower extremities. Previous research compared 2D versus 3D motion analysis using a variety of frame rates, making direct comparisons difficult. For example, Alahmari et al. [18] found no significant differences between 2D at 30 fps and 3D motion capture for measuring FPPA during single-leg landing, suggesting that although 2D at 30 fps is a practical instrument, it may not completely capture the complexity of multiplanar joint movements. Our results build on this by demonstrating that increasing the frame rate of 2D to 120 fps further enhances the measurement accuracy.

This study highlights the advantage of using higher frame rates in 2D motion analysis because they capture more frames per second, thereby reducing the likelihood of missing peak movement angles. This is crucial for accurately evaluating dynamic movements, where even slight timing variations can result in significant differences in measurements. Although certain studies have proposed specific frame rates for activities, such as walking or sprinting, these recommendations are often not experimentally validated, particularly in the context of high-intensity sports, such as football [22]. Therefore, further research is necessary to establish robust guidelines for optimal frame rates in 2D motion analysis, particularly for high-speed activities, such as cutting and jumping.

Our study findings have potential implications for both clinical and sports performance settings. High-speed cameras for 2D motion analysis substantially improve the accuracy of assessments, particularly for measuring knee valgus and trunk flexion angles during rapid movements. This enhanced precision is critical for identifying potential risk factors for lower extremity injuries, such as ACL tears. Healthcare professionals and sports scientists should incorporate high-speed cameras into their standard evaluation protocols to capture crucial kinematic data more effectively. Additionally, the notable discrepancies observed in LTF measurements emphasize the importance of closely monitoring trunk control during dynamic tasks such as side-cutting, because poor trunk control can exacerbate the risk of lower-limb injuries.

This study underscores the need for high-frame cameras (e.g., 120 fps) in sports and clinical settings to optimize the accuracy of kinematic assessments, specifically for dynamic movements such as single-leg landing and side-cutting, where lower frame rates may underestimate knee valgus and trunk motion. Sports and clinicians’ scientists should prioritize high-frame motion analysis for injury screening, rehabilitation, and return-to-play assessment, while recognizing that 30 fps may suffice for more stable movements such as hip adduction. This finding aligns with those of Alahmari et al. [18], specifically regarding the measurement of HADD using 2D motion analysis. Alahmari et al. [18] demonstrated strong correlations between 2D at 30 fps and 3D HADD during single-leg landing, showing that 2D motion capture at 30 fps provides reliable assessments of HADD. Similarly, our study found that HADD measurements were not significantly impacted by frame rate changes (30 fps vs. 120 fps), suggesting that HADD is a relatively stable frontal plane movement that can be accurately captured even at lower frame rates.

This study had several limitations that should be acknowledged. First, the study exclusively included male athletes, which limits the generalizability of the findings. Future research should include female participants to determine whether the effects of frame rate on kinematic measurements are consistent across sexes. Additionally, there was a significant disparity between the frame rates tested (30 and 120 fps), which may have overlooked the subtle effects of intermediate frame rates, such as 60 or 90 fps. Further research should investigate these intermediate frame rates to determine optimal camera speeds for various functional tasks. Finally, expanding the scope of tasks beyond single-leg landing and side-cutting, such as incorporating running, sprinting, or jumping, could broaden the applicability of the findings.

Further research is necessary to validate these findings across larger and more diverse participant samples, including both elite and recreational athletes of various ages and skill levels. Future studies should explore how different frame rates affect other important kinematic variables, such as hip internal rotation and ground reaction forces, which are critical for both injury prevention and performance enhancement. Expanding the range of movements analyzed beyond those examined in this study could provide a more comprehensive understanding of how frame rates influence biomechanical evaluations. Additionally, developing standardized guidelines for the appropriate use of specific camera frame rates in 2D motion analysis would enhance consistency and accuracy in both clinical assessment and sports performance monitoring.

## 5. Conclusions

Our findings demonstrated significant differences in kinematic measurements when using high-speed (120 fps) versus low-speed (30 fps) cameras, particularly for assessing FPPA and LTF during functional tasks. These findings suggest that high-speed cameras provide more accurate and detailed kinematic data, which are crucial for identifying risk factors related to lower-limb injuries. To confirm these findings, future research is recommended to replicate this study with larger and more diverse participant samples, including female athletes. Furthermore, examining the effects of intermediate frame rates (e.g., 60 fps or 90 fps) could help to detect the optimal resolution for various movement tasks. Developing standardized guidelines for frame rate selection in 2D motion analysis is essential to enhancing the consistency and applicability of biomechanical assessments in both clinical and sports settings.

## Figures and Tables

**Table 1:** Demographic characteristics of participants.

Variable	Mean ± SD
Age, years	24.37 ± 3.14
Height, cm	170.48 ± 65.68
Weight, kg	65.86 ± 6.13

**Table 2:** Comparison of frontal plane projection angle (FPPA), hip adduction angle (HADD), and lateral trunk flexion (LTF) for dominant and non-dominant leg during single-leg landing and for right leg during side-cutting using a 30 vs. 120 frames per second (fps) camera.

Task	Side	Angle	Frames per Second (fps; Mean ± SD)		Mean Difference (95% CI)	Cohen’s d	*p* Value
			30 fps	120 fps			
Single-leg landing	Dominant	FPPA	−7.87 ± 4.37	−10.53 ± 4.25	2.65 (0.76–4.55)	0.61	**0.008**
		HADD	86.26 ± 5.40	85.14 ± 5.61	1.11 (−0.51–2.70)	0.20	0.170
		LTF	11.56 ± 3.14	11.03 ± 3.17	0.52 (−1.05–2.10)	0.16	0.500
	Non-dominant	FPPA	−7.01 ± 5.16	−10.55 ± 4.96	3.53 (1.53–5.54)	0.70	**0.001**
		HADD	88.05 ± 1.02	86.98 ± 1.11	1.06 (−0.55–2.66)	1.00	0.188
		LTF	11.02 ± 0.47	12.19 ± 0.57	−1.16 (−2.44–0.10)	−2.23	0.069
Side-cutting	Right leg	FPPA	−7.93 ± 3.79	−10.11 ± 6.74	2.18 (0.43–3.93)	0.40	**0.016**
		HADD	92.85 ± 1.41	92.12 ± 1.60	0.73 (−0.98–2.43)	0.48	0.389
		LTF	5.26 ± 0.97	7.69 ± 1.42	−2.69 (−5.17–−0.22)	−2.20	**0.034**

Bold values indicate statistical significance at *p* < 0.05.

## Data Availability

The original data presented in this study are openly available in the Health & Medical Care Archive (ID: ICPSR-216062).

## Abbreviations

The following abbreviations are used in this manuscript:

ACL	Anterior cruciate ligament
ASIS	Anterior superior iliac spine
FIFA	Federation of International Football Association
fps	Frames per second
FPPA	Frontal plane projection angle
HADD	Hip adduction
LTF	Lateral trunk flexion
STROBE	Strengthening the Reporting of Observational Studies in Epidemiology
3D	Three-dimensional
2D	Two-dimensional
